# Changing Patterns of Human Campylobacteriosis, England and Wales, 1990–2007

**DOI:** 10.3201/eid1512.090280

**Published:** 2009-12

**Authors:** Iain A. Gillespie, Sarah J. O’Brien, Frederick J. Bolton

**Affiliations:** Health Protection Agency Centre for Infections, London, UK (I.A. Gillespie); University of Manchester; Manchester, UK (S.J. O’Brien); Regional Health Protection Agency Laboratory, Manchester (F.J. Bolton)

**Keywords:** Campylobacter, epidemiology, England, Wales, aging, bacteria, dispatch

## Abstract

To explore hypotheses for age-related changes in the incidence of *Campylobacter* infections in England and Wales during 1990–2007, we analyzed electronic laboratory data. Disease incidence was reduced among children, and the greatest increase in risk was for those >60 years of age. Risk factors for campylobacteriosis in the elderly population should be identified.

*Campylobacter* infection remains a major public health problem worldwide. The infection is unpleasant, although generally self-limiting, and most patients experience acute enteritis for 7–10 days (*1*). Approximately one tenth of patients with laboratory-confirmed cases require hospital treatment as a result of their illness (*2*), and a range of gastrointestinal, arthritic, and neurologic sequelae add to disease effects (*3*). Although food is likely the main source of transmission to humans, most human infections cannot be explained by recognized risk factors.

In the late 1970s, when the role of *Campylobacter* spp. in human gastrointestinal disease had been newly appreciated, the number of laboratory-confirmed infections in England and Wales began to rise; 8,956 cases were reported in 1980 and 33,234 in 1989 (Health Protection Agency, unpub. data). This increase was largely artifactual, reflecting increased scientific interest in, and testing for, *Campylobacter* spp. and improvements in media and methods for isolating them (*4*,*5*). The incidence continued to rise throughout the 1990s and peaked in 2000 at 58,236 cases. The reasons for this increase are unknown; further methodologic improvements or increased surveillance activity in that decade cannot fully explain it. Incidence rapidly decreased between 2000 and 2004 (from 57,674 to 44,294 cases; 24% decrease; Health Protection Agency, unpub. data), after which incidence increased for 3 consecutive years; provisional total was 51,758 cases in 2007 (Health Protection Agency, unpub. data). The reasons for these recent changes in incidence are again unknown. To explore hypotheses for changes in incidence related to age, we analyzed electronic laboratory data for *Campylobacter* infections reported in England and Wales from 1990 through 2007.

## The Study

Data on all *Campylobacter* isolates obtained from fecal or lower gastrointestinal tract samples, reported in England and Wales from 1990 through 2007, were extracted from the national laboratory database (LabBase) and stored in a Microsoft Access (Microsoft Corp., Redmond, WA, USA) database. Cases were assigned to 10-year age groups and to geographic areas on the basis of laboratory location (northern, mid-country, southern). The season was assigned on the basis of the earliest available specimen date (spring, March–May; summer, June–August; autumn, September–November; winter, December–February). Data on cases of cryptosporidiosis and nontyphoidal salmonellosis for the same period were extracted, grouped, and categorized and manipulated as above for comparative purposes. Denominator data for England and Wales for the same period were obtained from the Office for National Statistics. Data were analyzed in Microsoft Excel 2003 and Stata version 10 (StataCorp, College Station, TX, USA). Estimates of incidence per 100,000 population were calculated throughout; relative risks (RR) and 95% confidence intervals (CIs) were calculated as required.

**Figure 1:**
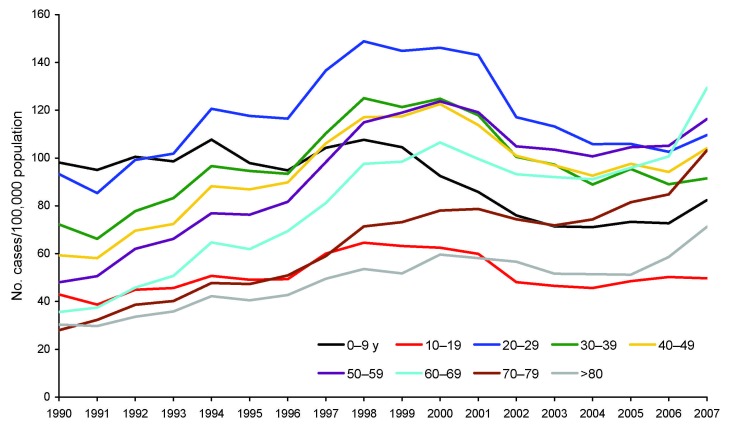
Incidence of laboratory-reported campylobacteriosis, England and Wales, by age group, 1990–2007.

**Figure 2:**
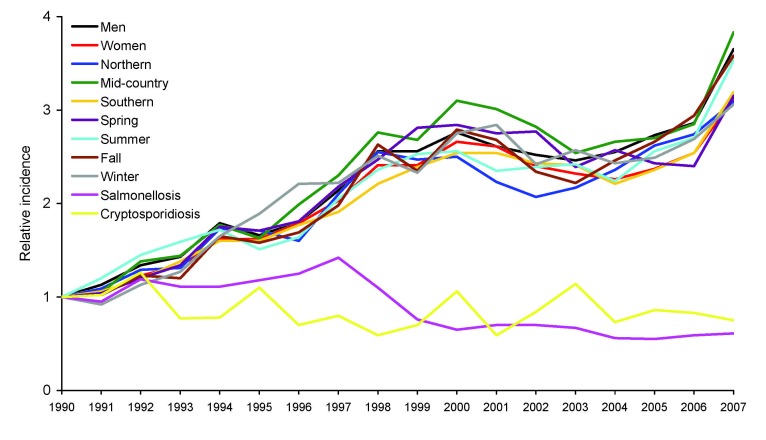
Relative incidence of campylobacteriosis by sex, region, and season, compared with rates of salmonellosis and cryptosporidiosis, among patients >60 years of age, England and Wales, 1991–2007. Northern, northwest, northeast, as well as Yorkshire and the Humber regions; mid-country, Wales, West Midlands, East Midlands, and East of England regions; southern, London as well as southeast and southwest regions. Salmonellosis includes nontyphoidal salmonellae, with age data available for 356,270 of 380,915 case-patients (94%); cryptosporidiosis includes age data for 76,462 of 79,808 case-patients (96%).

From 1990 through 2007 in England and Wales, 838,436 cases of *Campylobacter* infection were reported; patient age was available for 810,632 case-patients (96.7%). From 1990 through 1999, incidence increased in all age groups (Figure 1), but the increase was proportionate to increasing age (0–9 years of age RR 1.07 [95% CI 1.03–1.10]; 10–19 years RR 1.47 [95% CI 1.41–1.55]; 20–59 years RR 1.78 [95% CI 1.75–1.81]; >60 years RR 2.51 [95% CI 2.41–2.61]). From 2000 through 2004, incidence declined in all age groups. However, although the degree of decline was similar for those 0–9 years of age (RR 0.77 [95% CI 0.74–0.8]), 10–19 years (RR 0.73 [95% CI 0.70–0.76]), and 20–59 years (RR 0.75 [95% CI 0.74–0.76]), for patients >60 years of age, the degree of decline was significantly lower (RR 0.88 [95% CI 0.86–0.91]; χ^2^ p<0.001). Finally, although the incidence increased only moderately among those 10–19 years of age (RR 1.02 [95% CI 0.98–1.07]) and 20–59 years (RR 1.04 [95% CI 1.03–1.06]) from 2005 through 2007, greater increases were observed for those 0–9 years (RR 1.12 [95% CI 1.08–1.17]) and those >60 years (RR 1.33 [95% CI 1.29–1.36]). During the surveillance period, therefore, the incidence among patients >60 years of age, compared with the incidence among younger patients, increased markedly (RR 0.45 [95% CI 0.44–0.47] in 1990 to RR 1.17 [95% CI 1.15–1.19] in 2007). This effect was observed for campylobacteriosis in both sexes, in 3 geographic areas of England and Wales, and in all 4 seasons, but was not observed for nontyphoidal salmonellosis or cryptosporidiosis (Figure 2; Technical Appendix).

## Conclusions

We report a striking change in the population at risk for campylobacteriosis in England and Wales, which is independent of gender, geography, or season. The absence of a similar change in the age distribution of laboratory-reported salmonellosis or cryptosporidiosis from the same population suggests that this is unlikely to be a surveillance artifact.

*Campylobacter* infections are rarely typed beyond the genus level in England and Wales, so infections are routinely reported as *Campylobacter* species. Therefore, changes in the incidence of the various species that constitute this broad case definition could possibly explain some of the altered disease pattern reported. For example, infections caused by certain species (e.g., *C. fetus*) are more often associated with coexisting conditions that might occur more frequently in the elderly. This circumstance was unlikely to have affected the results of this study, however. First, isolation methods used in England and Wales favor the growth of *C. jejuni* and *C. coli* (*4*,*5*) over that of other species, including *C. fetus*. Furthermore, *C. fetus* normally causes systemic infections detected through blood culture, and the proportion of blood to fecal isolations of *Campylobacter* species in patients >60 years of age reported in England and Wales remained constant from 1990 through 1999 (275/58,139 fecal isolations; 0.47%), from 2000 through 2004 (185/44,349; 0.42%), and from 2005 through 2007 (135/31,637; 0.42%) (Health Protection Agency, unpub. data).

When disease incidence was ranked according to specific population group, children 0–9 years of age had the highest ranking in 1990, but by 2007, incidence for this age group ranked seventh of 9 age groups. Incidence among children <10 years declined most rapidly from 1998 to 2003. This finding led to the hypothesis that the introduction and increased utilization of the National Health Service’s NHS Direct (a 24-hour telephone, online, and interactive digital TV service, which provided health advice and information) at this time had a “triage effect” on those seeking care for children in this age group. The 2 events are correlated in time (initial NHS Direct pilot sites began taking calls in March 1998; by April 1999, 40% of the population of England had access, and by November 2000, the service was available throughout England and Wales [*6*]), NHS Direct has had a demonstrable negative effect on the use of general practice (*7*), and infants and young children are overrepresented among calls to NHS Direct about gastrointestinal conditions (*8*). The second Infectious Intestinal Disease study in England, currently under way (*9*), will provide further information upon which to assess this hypothesis, which is not readily testable by using laboratory data.

By far the most striking finding of this study is the emergence of older persons as the population most at risk for campylobacteriosis in England and Wales. Although the elderly were not the only group at risk in 2007 (because of increasing incidence), the overall trend singles them out as the main emerging at-risk group (the increase in other age groups requires continued monitoring, however). The pattern of infection in older patients is perhaps predictable, given the similar pattern for incidence of listeriosis in England and Wales since 2001 (*10*). As life expectancy increases in the United Kingdom, the number of persons living with chronic conditions is likely to increase; these factors suggest that the incidence of campylobacteriosis in older persons will continue to increase in the future. Therefore, risk factors for *Campylobacter* infection specific to older UK residents must be identified.

## Supplementary Material

Technical Appendix Relative incidence of Campylobacter infection in each year compared with that in 1990 among patients >60 years of age infected with selected gastrointestinal pathogens, England and Wales, 1991-2007

## References

[1] Skirrow MB, Blaser MJ. Clinical aspects of *Campylobacter* infection. In: Nachamkin I, Blaser MJ, editors. *Campylobacter*, 2nd ed. Washington: ASM Press; 2000. p. 69–88.

[2] Communicable Disease Surveillance Center. *Campylobacter* sentinel surveillance: the first year. Commun Dis Rep CDR Wkly [serial online]. 2000;11(35) [cited 2001 Aug 31]. Available from http://www.hpa.org.uk/cdr/archives/2001/cdr3501.pdf

[3] Vandenberg O, Skirrow MB, Butzler JP. *Campylobacter* and *Arcobacter*. In: Borriello SP, Murray PR, Funke G, editors. Topley & Wilson’s microbiology and microbial infections. Bacteriology, vol. 2, 10th ed. London: Hodder Arnold; 2005. p. 1541–62.

[4] Bolton FJ, Hutchinson DN, Parker G. Isolation of *Campylobacter*: what are we missing? J Clin Pathol. 1987;40:702–3. 10.1136/jcp.40.6.702-b 3301911 PMC1141074

[5] Bolton FJ, Hutchinson DN, Parker G. Reassessment of selective agars and filtration techniques for isolation of *Campylobacter* species from faeces. Eur J Clin Microbiol Infect Dis. 1988;7:155–60. 10.1007/BF01963069 3134202

[6] Boardman J, Steele C. NHS Direct—a telephone helpline for England and Wales. Psychiatr Bull. 2002;26:42–4. 10.1192/pb.26.2.42

[7] Munro J, Nicholl J, O'Cathain A, Knowles E. Impact of NHS direct on demand for immediate care: observational study. BMJ. 2000;321:150–3. 10.1136/bmj.321.7254.150 10894694 PMC27434

[8] Cooper DL, Smith GE, O'Brien SJ, Hollyoak VA, Baker M. What can analysis of calls to NHS direct tell us about the epidemiology of gastrointestinal infections in the community? J Infect. 2003;46:101–5. 10.1053/jinf.2002.1090 12634071

[9] Second UK–wide. community-based infectious intestinal diseases study. Health Protection Report., vol. 2, no. 21, May 23, 2008 [serial online] [cited 2008 Dec 19]. Available from http://www.hpa.org.uk/hpr/archives/2008/news2108.htm

[10] Gillespie IA, McLauchlin J, Grant KA, Little CL, Mithani V, Penman C, Changing pattern of human listeriosis, England and Wales, 2001–2004. Emerg Infect Dis. 2006;12:1361–6. 17073084 10.3201/eid1209.051657 PMC3294736

