# Supplementary material for: Changing Patterns of Human Campylobacteriosis, England and Wales, 1990–2007
Source: Emerg Infect Dis. 2009 Dec;15(12):2046–8. doi: 10.3201/eid1512.090280 (PMC3044522; doi:10.3201/eid1512.090280)
Supplement: Technical Appendix — Relative incidence of Campylobacter infection in each year compared with that in 1990 among patients >60 years of age infected with selected gastrointestinal pathogens, England and Wales, 1991-2007 [file 09-0280_appT-s1.pdf]

Publisher: CDC; Journal: Emerging Infectious Diseases
Article Type: Dispatch; Volume: 15; Issue: 12; Year: 2009; Article ID: 09-0280
DOI: 10.3201/eid1512.090280; TOC Head: Dispatch

Appendix Table. Relative incidence of *Campylobacter* infection in each year compared with that in 1990 among patients ≥60 years of age infected with selected gastrointestinal pathogens, England and Wales, 1991–2007

	Relative risk (95% confidence interval)										
	Campylobacter spp.										
	Sex		Area*			Season					
Year	Male	Female	Northern	Mid-country	Southern	Spring	Summer	Fall	Winter	Nontyphoidal salmonellae†	Cryptosporidiosis‡
1991	1.1 (1.1–1.2)	1.0 (1.0–1.1)	1.1 (1.0–1.2)	1.1 (1.0–1.1)	1.1 (1.0–1.1)	1.0 (0.9–1.1)	1.2 (1.1–1.3)	1.0 (0.9–1.1)	0.9 (0.8–1.0)	0.9 (0.9–1.0)	1.0 (0.8–1.3)
1992	1.3 (1.3–1.4)	1.2 (1.2–1.3)	1.3 (1.2–1.4)	1.4 (1.3–1.5)	1.2 (1.1–1.3)	1.2 (1.1–1.3)	1.4 (1.3–1.6)	1.2 (1.1–1.3)	1.1 (1.0–1.3)	1.2 (1.1–1.2)	1.3 (1.0–1.5)
1993	1.4 (1.3–1.5)	1.3 (1.3–1.4)	1.3 (1.2–1.4)	1.4 (1.3–1.6)	1.4 (1.3–1.5)	1.3 (1.2–1.5)	1.6 (1.5–1.7)	1.2 (1.1–1.3)	1.3 (1.1–1.4)	1.1 (1.1–1.2)	0.8 (0.6–1.0)
1994	1.8 (1.7–1.9)	1.6 (1.5–1.7)	1.7 (1.6–1.9)	1.8 (1.6–1.9)	1.6 (1.5–1.7)	1.7 (1.6–1.9)	1.7 (1.6–1.8)	1.6 (1.5–1.8)	1.7 (1.5–1.8)	1.1 (1.1–1.2)	0.8 (0.6–1.0)
1995	1.7 (1.6–1.8)	1.6 (1.5–1.7)	1.7 (1.6–1.9)	1.6 (1.5–1.8)	1.6 (1.5–1.7)	1.7 (1.6–1.9)	1.5 (1.4–1.6)	1.6 (1.5–1.7)	1.9 (1.7–2.1)	1.2 (1.1–1.2)	1.1 (0.9–1.4)
1996	1.8 (1.7–1.9)	1.8 (1.7–1.9)	1.6 (1.5–1.7)	2.0 (1.8–2.1)	1.8 (1.7–1.9)	1.8 (1.7–2.0)	1.6 (1.5–1.8)	1.7 (1.6–1.8)	2.2 (2.0–2.4)	1.3 (1.2–1.3)	0.7 (0.6–0.9)
1997	2.1 (2.0–2.3)	2.0 (1.9–2.1)	2.1 (1.9–2.3)	2.3 (2.1–2.5)	1.9 (1.8–2.0)	2.2 (2.0–2.4)	2.1 (1.9–2.2)	2.0 (1.8–2.1)	2.2 (2.0–2.4)	1.4 (1.4–1.5)	0.8 (0.6–1.0)
1998	2.6 (2.4–2.7)	2.4 (2.3–2.5)	2.6 (2.4–2.8)	2.8 (2.6–3.0)	2.2 (2.1–2.4)	2.5 (2.3–2.7)	2.4 (2.2–2.5)	2.6 (2.4–2.8)	2.5 (2.3–2.8)	1.1 (1.0–1.2)	0.6 (0.5–0.8)
1999	2.6 (2.4–2.7)	2.4 (2.3–2.5)	2.5 (2.3–2.7)	2.7 (2.5–2.9)	2.4 (2.2–2.5)	2.8 (2.6–3.1)	2.5 (2.4–2.7)	2.3 (2.2–2.5)	2.3 (2.1–2.6)	0.8 (0.7–0.8)	0.7 (0.6–0.9)
2000	2.8 (2.6–2.9)	2.7 (2.5–2.8)	2.5 (2.3–2.7)	3.1 (2.9–3.3)	2.5 (2.4–2.7)	2.8 (2.6–3.1)	2.6 (2.4–2.7)	2.8 (2.6–3.0)	2.8 (2.5–3.0)	0.6 (0.6–0.7)	1.1 (0.9–1.3)
2001	2.6 (2.5–2.8)	2.6 (2.5–2.8)	2.2 (2.1–2.4)	3.0 (2.8–3.2)	2.5 (2.4–2.7)	2.8 (2.5–3.0)	2.3 (2.2–2.5)	2.7 (2.5–2.9)	2.8 (2.6–3.1)	0.7 (0.7–0.7)	0.6 (0.5–0.8)
2002	2.5 (2.4–2.7)	2.4 (2.3–2.5)	2.1 (1.9–2.2)	2.8 (2.6–3.0)	2.4 (2.3–2.6)	2.8 (2.6–3.0)	2.4 (2.2–2.6)	2.3 (2.2–2.5)	2.4 (2.2–2.7)	0.7 (0.7–0.7)	0.8 (0.7–1.0)
2003	2.5 (2.3–2.6)	2.3 (2.2–2.5)	2.2 (2.0–2.3)	2.5 (2.4–2.7)	2.4 (2.3–2.6)	2.4 (2.2–2.6)	2.4 (2.3–2.6)	2.2 (2.1–2.4)	2.6 (2.3–2.8)	0.7 (0.6–0.7)	1.1 (0.9–1.4)
2004	2.6 (2.4–2.7)	2.3 (2.1–2.4)	2.4 (2.2–2.5)	2.7 (2.5–2.8)	2.2 (2.1–2.4)	2.6 (2.4–2.8)	2.2 (2.1–2.4)	2.5 (2.3–2.7)	2.4 (2.2–2.7)	0.6 (0.5–0.6)	0.7 (0.6–0.9)
2005	2.7 (2.6–2.9)	2.4 (2.2–2.5)	2.6 (2.4–2.8)	2.7 (2.5–2.9)	2.4 (2.2–2.5)	2.4 (2.2–2.6)	2.6 (2.4–2.7)	2.7 (2.5–2.9)	2.5 (2.3–2.7)	0.6 (0.5–0.6)	0.9 (0.7–1.1)
2006	2.9 (2.7–3.0)	2.5 (2.4–2.7)	2.7 (2.5–3.0)	2.8 (2.7–3.0)	2.5 (2.4–2.7)	2.4 (2.2–2.6)	2.7 (2.5–2.9)	2.9 (2.7–3.2)	2.7 (2.5–2.9)	0.6 (0.6–0.6)	0.8 (0.7–1.0)
2007	3.6 (3.4–3.9)	3.1 (3.0–3.3)	3.1 (2.9–3.3)	3.8 (3.6–4.1)	3.2 (3.0–3.4)	3.1 (2.9–3.4)	3.5 (3.3–3.8)	3.6 (3.3–3.9)	3.1 (2.8–3.4)	0.6 (0.6–0.7)	0.7 (0.6–0.9)

*Northern, northwest and northeast England as well as Yorkshire and the Humber regions; mid-country, Wales, West Midlands, East Midlands, and East of England regions; southern, London as well as southeast, and southwest regions.
†Age data available for 356,270/380,915 cases (94%).
‡Age data available for 76,462/79,808 cases (96%).
